# Coreceptors and Their Ligands in Epithelial γδ T Cell Biology

**DOI:** 10.3389/fimmu.2018.00731

**Published:** 2018-04-09

**Authors:** Deborah A. Witherden, Margarete D. Johnson, Wendy L. Havran

**Affiliations:** Department of Immunology and Microbiology, The Scripps Research Institute, La Jolla, CA, United States

**Keywords:** epithelial, γδ T cell, activation, costimulation, inhibition, epidermis, intestine, lung

## Abstract

Epithelial tissues line the body providing a protective barrier from the external environment. Maintenance of these epithelial barrier tissues critically relies on the presence of a functional resident T cell population. In some tissues, the resident T cell population is exclusively comprised of γδ T cells, while in others γδ T cells are found together with αβ T cells and other lymphocyte populations. Epithelial-resident γδ T cells function not only in the maintenance of the epithelium, but are also central to the repair process following damage from environmental and pathogenic insults. Key to their function is the crosstalk between γδ T cells and neighboring epithelial cells. This crosstalk relies on multiple receptor–ligand interactions through both the T cell receptor and accessory molecules leading to temporal and spatial regulation of cytokine, chemokine, growth factor, and extracellular matrix protein production. As antigens that activate epithelial γδ T cells are largely unknown and many classical costimulatory molecules and coreceptors are not used by these cells, efforts have focused on identification of novel coreceptors and ligands that mediate pivotal interactions between γδ T cells and their neighbors. In this review, we discuss recent advances in the understanding of functions for these coreceptors and their ligands in epithelial maintenance and repair processes.

## Introduction

The epithelial tissues are home to populations of T cells that function to protect the body from environmental pathogens and other insults. A major portion of T cells in many of these tissues expresses the γδ T cell antigen receptor (TCR) (1). The importance of these cells to homeostasis and wound repair has been evident for several years and exemplified by studies in skin, intestine, and lung (2–9). An absence of epithelial-resident γδ T cells in these tissues results in dysregulation of the epithelium, more severe damage or disease, and a delay in repair processes (2, 6, 8, 10, 11). Constant communication between resident γδ T cells and their neighboring epithelia is crucial for homeostasis and repair processes following damage or disease. Recent studies have begun to define the role of distinct molecular interactions in the rapid and localized response of epithelial-resident γδ T cells to tissue injury, yet much of the triggers and sequence of events remain a mystery.

## Epithelial Tissues

The resident T cell population in the epidermal layer of the murine skin is a highly dendritic γδ T cell termed dendritic epidermal T cell (DETC). DETC express a canonical Vγ3Vδ1 TCR [nomenclature according to Garman et al. (12); alternative nomenclature Vγ5Vδ1 (13)] and make numerous contacts with surrounding epithelial cells, in particular keratinocytes and Langerhans cells. Under homeostatic conditions, their individual dendrites extend between surrounding cells allowing for constant contact with numerous adjacent cells. This feature allows for regulated interactions between both cell surface receptors and soluble molecules facilitating homeostasis in the skin and allowing for rapid repair following damage or disease. Although differing in T cell composition, human epidermis also contains resident T cells that make crucial contributions to wound repair (9). As such, it is reasonable to suggest that similar mechanisms of communication exist in human epidermis.

The intestinal mucosal barrier is also occupied by resident T cells. These T cells are termed intraepithelial lymphocytes (IEL) that, as their name suggests, are found residing between epithelial cells and include subsets of both αβ and γδ T cells. The intestinal γδ T cell subset expresses predominantly a Vγ5 TCR [alternative nomenclature Vγ7 (13)] that is able to pair with a number of different Vδ chains. Although not dendritic like γδ T cells in the skin, γδ IEL are able to make contact with multiple epithelial cells through active migration within the intestinal epithelium. This gives a single γδ IEL the ability to surveil large areas of epithelium (14, 15) and defend against pathogenic assault (16). Although not as clearly defined, resistance to infection and repair from damage in the lung also relies on resident γδ T cells (3, 11, 17–20), again likely through numerous contacts with surrounding cells (21).

Continual interaction with neighboring epithelia is thus required for epithelial γδ T cells to perform their functions in homeostasis, resistance to infection, and damage repair. While the importance of the TCR is clear (22–25), it is becoming evident that additional distinct molecular interactions drive these functions of epithelial γδ T cells. Discussion of some of these interactions (Table 1) will be the focus of the remainder of this review.

**Table 1:** Non-TCR receptor–ligand pairs in epithelial γδ T cell function.

γδ T cell	Epithelial cell	Species	Function	Reference
Junctional adhesion molecule-like	Coxsackie and adenovirus receptor	Mouse and human	Costimulation	(64, 65)
NKG2D	MICA/MICB	Human	Costimulation	(71–74, 78, 79)
	Rae1, H60c, MULT-1	Mouse		
?	Skints	Mouse	Activation	(81)
	Butyrophilins	Mouse/human	Activation	(82)
CD200R	CD200	Mouse	Inhibition	(85)
CD94/NKG2	HLA-E	Human	Inhibition	(86–89)
	Qa-1	Mouse		
G protein-coupled receptor 55	L-α-lysophosphatidylinositol	Mouse	Inhibition	(15)
Lymphocyte function-associated antigen-1	Intercellular adhesion molecule 1	Mouse and human	Adhesion	(26, 27)
E-cadherin	?	Mouse	Adhesion	(42–44, 48, 49)
αEβ7	E-cadherin	Mouse	Adhesion	(45–50)
CD100	Plexin B2	Mouse	Morphology/migration	(58–60)
Aryl hydrocarbon receptor	?	Mouse	γδ T cell maintenance	(55–57)
TLR 2, 4, 9	?	Mouse	?	(83)
?	CD98hc	Mouse	?	(53)
CCR4		Mouse	γδ T cell maintenance	(54)

*?, unknown*.

## Adhesion

The maintenance of epithelial-resident γδ T cells within the epithelium is known to involve adhesion through integrin and cadherin-mediated interactions. Expression of these molecules is also modulated in response to epithelial damage suggesting their functions may extend beyond maintenance to roles in repair processes as well.

Intercellular adhesion molecule 1 (ICAM-1), also known as CD54, is a membrane-bound adhesion molecule that is a ligand for leukocyte-expressed lymphocyte function-associated antigen-1 (LFA-1). This protein is well known to recruit leukocytes to sites of inflammation, but its interaction with tissue-resident γδ T cells is less understood. ICAM-1 is upregulated by the corneal epithelium following wounding and is required for γδ T cell recruitment to the site of damage in an LFA-1-dependent process (26). While ICAM-1 is also upregulated by endothelial cells and keratinocytes following wounding (27), and ICAM-1-deficient mice are known to exhibit delayed wound repair (27, 28), it is unknown whether the protein plays any role in DETC-mediated epithelial repair. ICAM-1 has also been shown to be important in shaping the gut lymphocyte populations, with ICAM-1-deficient mice displaying a relatively higher proportion of γδ T cells and a lower proportion of αβ T cells, though the biological effects of this population shift are unclear (29). Interestingly, the effect of ICAM-1/LFA-1 interactions on γδ T cells is not limited to leukocyte migration. Costimulation of peripheral mouse γδ T cells through TCR and LFA-1 was demonstrated to trigger apoptosis of these cells (30, 31), in contrast to the proliferative response observed in αβ T cells (30). However, ICAM-1/LFA-1 interaction has been shown to be involved in peripheral γδ T cell recognition of tumor cells and subsequent cytolytic response (32–36), so the outcome appears to be context dependent. While the majority of this work has focused on γδ T cell recognition of target cell-expressed ICAM-1, it should be noted that γδ T cells also express ICAM-1 (37). Blocking ICAM-1 expressed on the epithelial-associated Vδ1 T cell population has been reported to reduce cytotoxicity against myeloma cells (34). Studies in αβ T cells have shown ICAM-1 to be a costimulatory molecule promoting proliferation, IL-2 and IFNγ secretion, phosphatidylinositol-3 kinase activation, and a shift toward a memory phenotype (38–40). However, it remains to be seen whether epithelial-resident γδ T cells also have the ability to receive costimulatory signals through ICAM-1, and what the effects of LFA-1 engagement are in this population.

E-cadherin is an adhesion molecule that supports adhesion between keratinocytes (41). Interestingly, DETC also express E-cadherin as well as another E-cadherin ligand, αEβ7. Following wounding, DETC downregulate expression of E-cadherin, but maintain their level of expression of αEβ7 (42–44). *In vitro* and *in vivo* studies have demonstrated a role for αEβ7 in DETC activation with possible functions in adhesion and epidermal retention, dendrite anchoring, morphology and motility, cytotoxicity and costimulation (22, 45–47). In contrast, DETC-expressed E-cadherin functions as an inhibitory receptor for DETC activation (47). Murine intestinal IEL also express both E-cadherin and αEβ7 (48, 49), and αEβ7 is expressed on most γδ T cells in the bleomycin-induced mouse model of lung fibrosis (50), suggesting similar functions for these adhesion molecules on γδ T cells in other epithelial sites. Furthermore, the expression of both E-cadherin and αEβ7 on fetal thymic precursors of DETC (43, 44) indicates that inhibitory and costimulatory signals, respectively through these molecules may also influence thymic development and maturation of DETC precursors. This is further supported by the observation of diminished DETC numbers in the epidermis of αE deficient mice (46), although thymic populations were not directly analyzed in this study.

CD98hc is an amino acid transporter that associates with both cadherins and β1 integrins (51, 52). As such, it is perhaps not surprising that it too has been implicated in the regulation of skin homeostasis and wound healing (53), although it is unknown whether this involves direct interaction of CD98hc with DETC. In addition to adhesive interactions, the chemokine receptor CCR4 has been shown to be important for DETC retention in the epidermis (54). Additionally, the aryl hydrocarbon receptor (AhR) transcription factor is essential for maintaining both DETC and IEL in their respective tissues (55–57), although just how AhR signals lead to tissue retention of DETC and IEL, and whether AhR plays a role in epithelial γδ T cell activation and the wound repair process, is unknown.

## Morphology and Migration

γδ IEL actively migrate within the intestinal epithelium and this migration is dependent on occludin expression in both IEL and the epithelium (14). In contrast, DETC in the epidermis are sessile under homeostatic conditions, communicating with surrounding keratinocytes through their numerous dendritic processes. Upon keratinocyte damage, DETC rapidly pull back these processes and adopt a more rounded morphology (6). Interestingly, downregulation of E-cadherin in keratinocytes can contribute to this rounding either through disruption of E-cadherin-mediated homophilic binding and/or αEβ7 integrin-mediated heterophilic binding (45).

In addition, binding of the semaphorin, CD100, to one of its ligands, Plexin B2, contributes to the DETC rounding response through activation of ERK kinase and cofilin (58). In the absence of CD100, the DETC rounding response to keratinocyte damage is delayed resulting in subsequent delayed wound closure (58). It has been suggested that the rounding of DETC permits them to migrate within the epidermis during wound repair, yet this remains to be demonstrated. Interestingly, in the intestinal epithelium, where IEL are in constant motion, CD100-plexin B2 interactions still play an important role as CD100-deficiency results in more severe damage as well as delayed repair in a mouse model of DSS-induced colitis (59). Similarly, a role for CD100 in lung allergic inflammation has been described (60). Whether CD100 is involved in γδ T cell migration in these epithelial tissues is yet to be determined.

## Activation

To become fully activated, αβ T cells require engagement of molecules in addition to the TCR, such as CD4, CD8, and CD28 together with other costimulatory and adhesion molecules. Unlike αβ T cells, epithelial-resident γδ T cells do not express CD4, CD8 (although the CD8aa homodimer is expressed by some γδ IEL), or CD28 (61), however, a number of other molecules have recently been described to participate in the activation of these cells.

Striking similarities between CD28 and the junctional adhesion molecule-like (JAML) (62–64) suggest that JAML may play the role of primary costimulator for epithelial-resident γδ T cells through interaction with its ligand coxsackie and adenovirus receptor (CAR) (64, 65), expressed on epithelial cells. Like CD28 on αβ T cells, JAML is able to induce proliferation and cytokine production in epithelial γδ T cells. This response is mediated through PI3K which is recruited to JAML following CAR ligation (63). The PI3K binding motif in CD28 (66), similarly mediates delivery of a costimulatory signal. Although JAML expression has been demonstrated on activated peripheral γδ T cells, a population of activated CD8^+^ αβ T cells and other cell types of both the innate and adaptive arms of the immune system, including neutrophils, monocytes, and some memory T cells (64, 65, 67), the function of JAML as a costimulatory molecule appears confined to the epithelial subsets of γδ T cells.

Blocking of JAML-CAR costimulation *in vivo* impairs DETC activation and delays wound closure (64), demonstrating the importance of this interaction to DETC function. Just how this interaction might function in response to other perturbations to the skin, such as infection or malignancy, is unknown. A parallel role in IEL activation in the mouse intestine (64) is suggested by the similarity in expression patterns of JAML and CAR in the intestine. Whether this costimulatory pair also functions in human skin and intestinal T cell activation and tissue repair is still not known.

The NKG2D receptor (discussed in detail elsewhere) is an activating receptor expressed on NK, γδ, and CD8^+^ T cells (10, 68–70). In the mouse epidermis, NKG2D is expressed on DETC and ligation to its ligands Rae-1, H60, and MULT-1 on keratinocytes activates DETC (10). A reliance on PI3K signaling has been demonstrated, however, whether activation through NKG2D also requires simultaneous TCR stimulation or can stimulate DETC directly is somewhat controversial (71–75). Nevertheless, the importance of NKG2D signaling in epithelial γδ T cells has been demonstrated in models of wound healing, carcinogenesis, and contact hypersensitivity responses (72, 76, 77). Whether the difference in T cell receptor requirement for NKG2D-mediated DETC activation is due to differences in the induced ligand resulting from the type of damage elicited, is unclear at this time. In humans, there is evidence to suggest that recognition of MIC by Vδ1 expressing intestinal epithelial T cells (76, 78, 79) can either be direct, *via* the TCR, through NKG2D, or sequentially using both molecules (80). This idea, however, requires experimental confirmation.

It is increasingly evident that additional molecules are also important for the activation of epithelial-resident γδ T cells. A recent analysis of defective wound healing in aged mice highlighted the importance of Skint molecules (to be reviewed in detail elsewhere) in DETC activation and epidermal re-epithelialization (81). A role for the closely related butyrophilin (Btnl) molecules in the activation of intestinal γδ T cells in both mice and humans has recently been demonstrated (82). In addition, other molecules, such as toll-like receptors 2, 4, and 9 have been shown to be upregulated on γδ T cells following skin injury (83), suggesting a role in their activation, however, a precise function has yet to be defined.

## Inhibition

The role of inhibitory signals in the control of αβ T cell activation is well established. Emerging evidence points to similar signals playing an important role in regulating the activation of epithelial-resident γδ T cells. The transmembrane glycoprotein CD200 expressed on keratinocytes has been implicated in the protection of hair follicles from autoimmune attack (84). Interestingly, resting DETC express low levels of the CD200-receptors 1, 2, and 3, but expression of CD200R1 is increased following activation *in vitro*. In functional assays, ligation of DETC-expressed CD200R with immobilized CD200 inhibits DETC proliferation and cytokine secretion highlighting an important role for CD200–CD200R interactions in the control of DETC activation (85). How this interaction may function during wound repair is unknown.

Inhibitory receptors expressed by NK cells are also found on γδ T cells, and appear to have similar inhibitory functions on these cells (86). The Ly49E and CD94/NKG2 receptors are expressed on mature fetal thymic DETC as well as those residing in the epidermis (86). DETC do not express other members of the Ly49 family. DETC cytotoxicity is inhibited by ligation of CD94/NKG2 and monoclonal antibody cross-linking of CD94/NKG2 prevents mature DETC thymocytes from killing FcγR^+^ target cells demonstrating a role for CD94/NKG2 as an inhibitory receptor on DETC (86). Just how these and other inhibitory interactions may function in epithelial wound repair processes warrants further investigation.

A recent report has identified an inhibitory role for G protein-coupled receptor 55 (GPR55) on intestinal IEL (15). GPR55 is highly expressed on γδ IEL and more modestly on αβ IEL and intestinal dendritic cells. Through interaction with its receptor L-α-lysophosphatidylinositol expressed on intestinal epithelial cells, GPR55 regulates the interaction between IEL and the epithelium and inhibits the accumulation of GPR55^+^ cells in the small intestine. Analysis of GPR55-deficient animals revealed increased γδ IEL migration within, and retention in, the small intestine, and enhanced IEL-epithelial cell crosstalk (15). Although the precise inhibitory role of GPR55 in the intestine is yet to be determined, Sumida et al. (15) propose that it may constrain IEL movement in the epithelium to allow normal epithelial cell functions to proceed.

## Conclusion

By analogy with skin, gut, and lung, the existence of a resident γδ T cell population in all epithelial barrier tissues implies a crucial function for these cells throughout the body. An increasing number of receptor-ligand pairs are being identified as vital for the homeostasis and repair functions of these resident γδ T cells. An understanding of the precise mechanisms of action of these various molecules in the crosstalk between T cells and their adjacent epithelial cells will help to elucidate their roles throughout the epithelia.

## Author Contributions

DW, MJ, and WH all contributed to the writing of the manuscript.

## Conflict of Interest Statement

The authors declare that the research was conducted in the absence of any commercial or financial relationships that could be construed as a potential conflict of interest.
